# An Unusual Case of Stevens-Johnson/Toxic Epidermal Necrolysis Overlap Syndrome in HER2 (Human Epidermal Growth Factor Receptor 2)-Positive Breast Cancer Patient Treated With Docetaxel

**DOI:** 10.7759/cureus.37590

**Published:** 2023-04-14

**Authors:** Gelan Shamloul, Mehir Desai, Nicole Laslett

**Affiliations:** 1 Oncology, Philadelphia College of Osteopathic Medicine, Philadelphia, USA; 2 Internal Medicine, Christiana Care Health System, Newark, USA; 3 Oncology, Christiana Care Health System, Newark, USA

**Keywords:** steven-johnson syndrome, locally advanced breast cancer, sjs/ten, scorten, her2-positive, toxic epidermal necrolysis (ten)

## Abstract

Stevens-Johnson syndrome (SJS) and toxic epidermal necrolysis (TEN) are rare but life-threatening drug-induced hypersensitivity reactions existing as a disease continuum based on the area of skin detachment. Following three cycles of treatment with docetaxel, a 60-year-old female with early-stage human epidermal growth factor receptor 2 (HER2)-positive breast cancer presented to the hospital with a flu-like illness and black crusting of the bilateral orbits, navel, and perianal region. Nikolsky sign was positive, and the patient was subsequently transferred to a specialized burn center for treatment of SJS/TEN overlap syndrome. There are a small number of cases documenting SJS/TEN following docetaxel administration in cancer patients.

## Introduction

Stevens-Johnson syndrome (SJS) and toxic epidermal necrolysis (TEN) exist as a spectrum of mucocutaneous hypersensitivity reactions with a mortality rate of up to 30% [1]. SJS/TEN most commonly presents as an adverse drug reaction; however, infectious agents, immunotherapy, and tamoxifen have also been implicated [2]. Approximately 25% of cases are idiopathic [1]. Genetic predisposition in carriers of different human leukocyte antigens (HLA)-B has been demonstrated [2]. This syndrome develops following a prodromal period of flu-like symptoms and manifests as painful, targetoid macules that coalesce with resultant skin desquamation. Erosions often develop in the eyes, oral, and genital mucosa. Treatment is largely supportive, with aggressive fluid and electrolyte replacement, nutritional support, and analgesia [2]. Currently, the evidence in support of systemic corticosteroid or intravenous immune globulin (IVIG) therapy is limited.

## Case presentation

**Figure 1:**
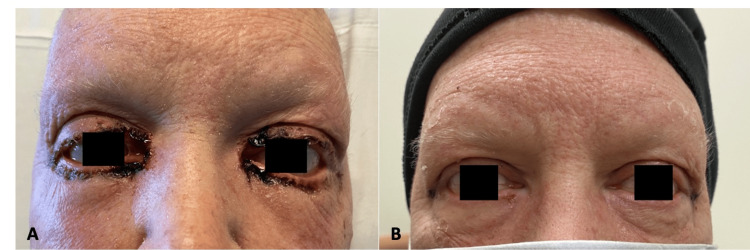
Ocular involvement Ocular involvement (a) at admission and (b) one week after discharge from the burn unit.

**Figure 2:**
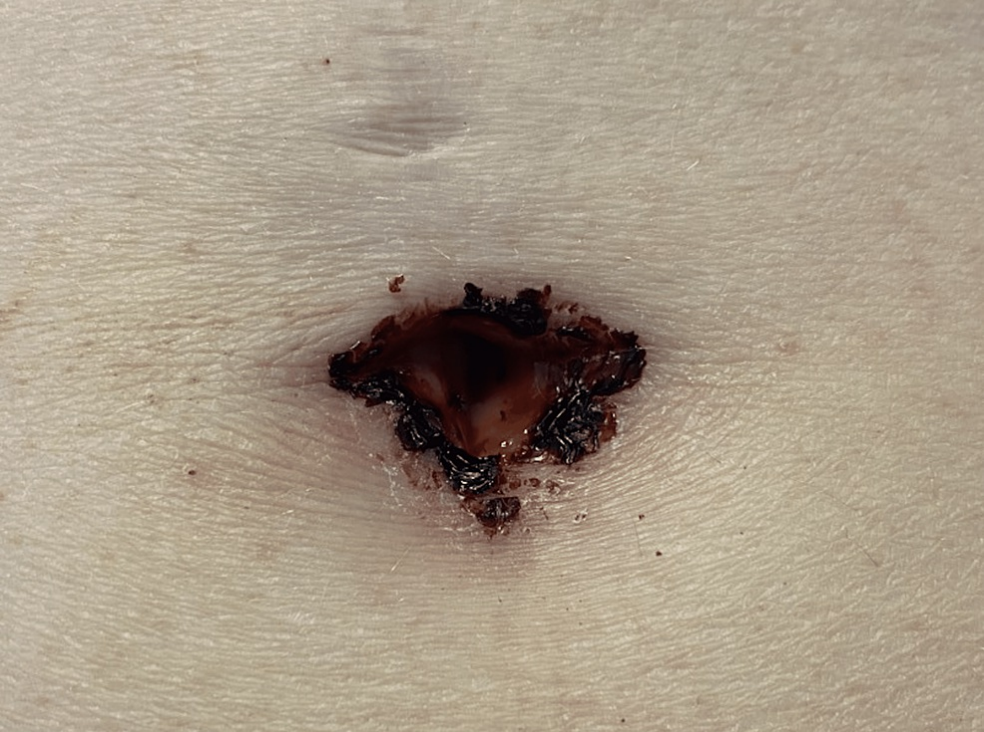
Hemorrhagic ulceration of the navel at the time of presentation.

**Figure 3:**
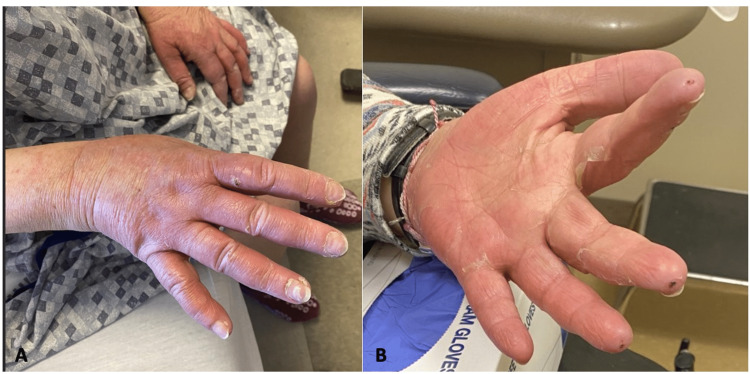
Bilateral hand edema and erythema Bilateral hand edema and erythema (a) at admission and (b) continued skin peeling one week after discharge from the burn unit.

A 60-year-old female with an extensive past medical history of atrial fibrillation, transient ischemic attack, hyperlipidemia, diabetes mellitus, mitral valve disorder, pulmonary hypertension, and chronic kidney disease was diagnosed with invasive mammary cancer of the left breast. Home medications included Eliquis, aspirin, metformin, carvedilol, gabapentin, rosuvastatin, and spironolactone. She was accepted into a clinical trial of four cycles of 75 mg/m^2^ docetaxel (Taxotere), in conjunction with the monoclonal antibodies 8 mg/kg trastuzumab (Herceptin) and 840 mg pertuzumab (Perjeta). Following the third cycle of treatment, the patient presented to the hospital after developing black, hemorrhagic crusting and purulent drainage from bilateral orbits (see Figure 1a), navel (Figure 2), and perianal region. Her bilateral hands and feet were pink and edematous with areas of desquamation (Figure 3a). This was accompanied by generalized fatigue, severe pain, weakness, and decreased oral intake. On presentation, labs showed a white blood cell (WBC) of 27.3 (4.5-10 thou/cmm). Wound cultures from the eyes and navel grew *Streptococcus agalactiae* (group B Strep) and moderate *Staphylococcus aureus*. She was treated in the emergency department with intravenous (IV) fluids, ceftriaxone, and doxycycline for presumed bacterial infection. CT scan of the head and orbits was negative for acute pathology. Following evaluation by dermatology, 80 mg of IV methylprednisolone was administered for suspected mucosal-limited SJS/TEN overlap syndrome. Ophthalmology was consulted and lysed adhesions formed between the upper and lower eyelids. No scarring of the conjunctiva or cornea was observed. A Severity-of-Illness Score for Toxic Epidermal Necrolysis (SCORTEN) score of three was calculated placing the patient at a 35.3% risk of mortality. She was transferred to a specialized burn center where she began to develop sloughing of the bilateral hands and wrists. A biopsy was not performed. She was treated with erythromycin (Romycin) ophthalmic ointment, vitamin A and D ointment to the orbits and naval, and zinc oxide 20% ointment to the perianal region. One week following discharge, the patient’s eyes were almost fully healed, and her vision remained unaffected (Figure 1b). The skin of her hands, legs, and perianal region continued to peel one week post-discharge (Figure 3b).

## Discussion

Stevens-Johnson syndrome (SJS) and toxic epidermal necrolysis (TEN) are life-threatening mucocutaneous hypersensitivity reactions that exist as a disease continuum [1]. SJS is characterized as <10% body surface area (BSA) of skin detachment, >30% is termed TEN, and between 10% and 30% is deemed SJS/TEN overlap [2]. Early recognition and appropriate treatment are critical as SJS/TEN has been associated with mortality rates of up to 30% [3]. The Severity-of-Illness Score for Toxic Epidermal Necrolysis (SCORTEN) can be utilized to predict SJS/TEN mortality (see Table 1) [4].

**Table 1:** The Severity-of-Illness Score for Toxic Epidermal Necrolysis (SCORTEN) The Severity-of-Illness Score for Toxic Epidermal Necrolysis (SCORTEN) is a predictor of mortality [4]. One point is awarded for each risk factor.

Risk factors (1 point each)		SCORTEN (points)	Mortality (%)
Age	≥40 years old	0-1	3.2
Heart rate	≥120 bpm	2	12.1
Cancer or hematologic malignancy	Any	3	35.3
Body surface area (BSA)	>10%	4	58.3
Blood urea nitrogen (BUN)	>10 mmol/L	≥5	90
Bicarbonate	<20 mmol/L (20 mEq/L)		
Glucose	>224 mmol/L (>252 mg/dL)		

The incidence of SJS is recently reported as 3.96-5.30/1,000,000 in adults and 6.3/100,000 for the pediatric population [5]. TEN was reported to occur in 0.4-1.45/1,000,000 adults and 0.5/100,000 children [5]. SJS/TEN is primarily an adverse drug reaction, with sulfonamide antibiotics, anti-epileptics (carbamazepine, lamotrigine, phenytoin), non-steroidal anti-inflammatory drugs, nevirapine, and allopurinol being associated with the highest risk [2,6]. Infectious agents have also been implicated, mainly by mycoplasma, herpes simplex virus, and cytomegalovirus [2]. Few cases have been reported following cancer treatments, including vemurafenib, ipilimumab, tamoxifen, nivolumab, and pembrolizumab [1].

SJS/TEN presents initially as erythematous, painful, and targetoid macules beginning on the trunk and extending to the face and limbs [5]. The macules later coalesce, and vesicles and bullae may develop [1]. Tangential pressure to the skin causes epidermal detachment, termed a positive Nikolsky sign [5]. Typically, the skin manifestations follow a flu-like prodrome with malaise, fever, sore throat, and conjunctivitis. The rash rarely affects the palms, soles, or scalp. Ocular, oral, and genital mucocutaneous involvement is common, causing erythema and erosions [5]. Epidermal detachment places patients at risk of secondary bacterial infection. Other complications reported include dehydration, hepatitis, sepsis, and pneumonia [5].

SJS/TEN is a type IV hypersensitivity reaction. A strong CD8+ T-cell response induces apoptosis of keratinocytes via the Fas-Fas ligand and the perforin/granzyme pathways [2]. The pathogenesis is still not fully understood, but there is increasing evidence of genetic predisposition playing a major role. A relationship between carbamazepine-induced SJS/TEN in the Han Chinese, Thai, Malaysian, and Indian populations with HLA-B 15:02 has been established [2]. HLA-B 57:01 was linked to SJS/TEN in HIV patients following treatment with abacavir [2]. In European and Asian populations, carriers of HLA-B 58:01 were increased for allopurinol-induced SJS/TEN [1].

Docetaxel (Taxotere) is a taxane chemotherapeutic agent that works by disrupting microtubule function and thus blocking cell cycle progression [7]. The Neosphere and TRYPHENA trials helped establish the role of pertuzumab in the neoadjuvant setting in combination with trastuzumab and chemotherapy for early-stage human epidermal growth factor receptor 2 (HER2) breast cancer. Given the modest benefit seen with pertuzumab in the APHINITY trial, the benefit of pertuzumab may be more discernable in patients who de-escalate therapy, such as in the Compass HER2 trial, where our patient was enrolled. A handful of cases of SJS/TEN following docetaxel therapy have been reported. These include SJS in a male patient following treatment with docetaxel and cyclophosphamide for breast cancer and docetaxel plus leuprolide for prostate cancer [8,9].

Supportive care is the mainstay of therapy for SJS/TEN, as effective treatment has not been well-defined. This includes aggressive fluid and electrolyte replacement, nutritional support, and proper analgesia. Ocular involvement is treated with corticosteroid drops and antibiotic eye drops or ointment [1]. Intravenous steroids and IVIG are commonly used in practice; however, there is limited evidence supporting improved outcomes with systemic therapy [1,10].

## Conclusions

In summary, SJS and TEN occur on a spectrum with 10%-30% BSA involvement deemed SJS/TEN overlap syndrome. Cases of SJS have been presented following docetaxel administration in combination with other chemotherapeutic agents, including cyclophosphamide and trastuzumab. This case highlights the importance of adequately assessing the skin, especially the mucocutaneous membranes, in patients being treated with docetaxel in order to detect early signs of a hypersensitivity reaction.
